# The Learning Curve for Transoral Endoscopic Thyroidectomy Without Neuromonitoring: Analysis of First 103 Cases From India

**DOI:** 10.1002/oto2.70142

**Published:** 2025-07-18

**Authors:** Sanjay Kumar Yadav, Goonj Johri, Saket Shekhar, Pawan Agarwal, Dhananjaya Sharma

**Affiliations:** ^1^ Department of Surgery Breast and Endocrine Surgery Unit, NSCB Medical College Jabalpur India; ^2^ Princess Anne Hospital University Hospital Southampton Southampton UK; ^3^ Department of Community Medicine and Biostatistics ESIC Bihta Patna India; ^4^ Department of Plastic Surgery NSCB Medical College Jabalpur India; ^5^ Department of Surgery NSCB Medical College Jabalpur India

**Keywords:** CUSUM analysis, hemithyroidectomy, India, intraoperative neuromonitoring (IONM), learning curve, low‐resource settings, minimally invasive surgery, operative time reduction, recurrent laryngeal nerve (RLN) palsy, scarless thyroid surgery, surgical outcomes, transoral endoscopic thyroidectomy (TOETVA), thyroid nodule

## Abstract

**Objective:**

Transoral endoscopic thyroidectomy via the vestibular approach (TOETVA) offers a scarless alternative to conventional thyroidectomy. Most studies incorporate intraoperative neuromonitoring (IONM), which may be unavailable in resource‐limited settings. We evaluated the learning curve, feasibility, and safety of TOETVA without IONM.

**Study Design:**

Retrospective.

**Setting:**

A retrospective analysis of 103 patients undergoing hemithyroidectomy by TOETVA between February 2020 and January 2025 was conducted at a tertiary care center in central India.

**Method:**

Learning curve assessment was performed using Cumulative Sum (CUSUM) analysis, and outcomes were compared between phase 1 (cases 1‐50) and phase 2 (Cases 51‐103). Statistical analyses included independent *t* tests for continuous variables and chi‐square tests for categorical variables (*P* < .05).

**Results:**

Mean operative time significantly decreased from 185 ± 24 minutes in phase 1 to 105 ± 12.95 minutes in phase 2 (*P* < .001), with proficiency achieved after 50 cases. Nodule size was larger in phase 2 (4.5 ± 2.3 cm vs 3.0 ± 1.0 cm, *P* = .003). The conversion rate was 4.9%, with no permanent recurrent laryngeal nerve palsy. Hoarseness of voice and seroma rates remained unchanged (*P* = 1.00), whereas hospital stay significantly decreased (*P* < .001).

**Conclusion:**

TOETVA without IONM is feasible and safe, demonstrating a well‐defined learning curve with low complication rates. These findings support its adoption in low‐resource settings.

Transoral endoscopic thyroidectomy via the vestibular approach (TOETVA) is a widely accepted scarless alternative to conventional thyroidectomy, offering superior cosmetic outcomes and patient satisfaction.^1^, ^2^, ^3^, ^4^ However, its adoption is hindered by a steep learning curve.^5^, ^6^, ^7^, ^8^ Intraoperative neuromonitoring (IONM) is often used to facilitate recurrent laryngeal nerve (RLN) identification and reduce nerve‐related complications; however, its high cost and limited availability pose challenges in resource‐constrained settings.^9^, ^10^, ^11^, ^12^, ^13^ We evaluated the learning curve for TOETVA without IONM in India, analyzing operative time, complication rates, and proficiency milestones.

## Methods

### Study Design and Setting

We retrospectively analyzed the first 103 consecutive cases of TOETVA without IONM, performed by a single surgeon at a tertiary teaching hospital in central India. The study period extended from February 2020 to January 2025, during which all eligible patients undergoing TOETVA were included. Ethical approval for the study was obtained from the Institutional Ethics Committee of NSCB Medical College, Jabalpur (IEC/2023/6497), and all patients provided informed consent before surgery for their data to be analyzed in any future research.

### Patient Selection

#### Inclusion Criteria

- Patients undergoing TOETVA with a preoperative diagnosis of a benign nodule.
- Nodule size ≤6 cm for benign lesions.
- No prior history of neck surgery or radiation exposure.

#### Exclusion Criteria

- Patients with thyroid cancer.
- Presence of retrosternal goiter.
- Patients undergoing total thyroidectomy.

### Surgical Technique

All procedures were performed using a standardized three‐port transoral endoscopic approach using conventional laparoscopic instruments under general anesthesia with nasal endotracheal intubation.^3^, ^14^ We incorporated specific modifications to the standard technique to make the procedure easier and safer.^15^, ^16^ The RLN was identified visually without the use of IONM in all patients.^16^ A suction drain was placed in selected cases, and the incisions were closed with absorbable sutures.

### Data Collection

Demographic, clinical, and perioperative data were collected from medical records, including patient demographics (age, sex), nodule characteristics (size, laterality, and histopathology), operative parameters (operative time, conversion to open surgery), and postoperative outcomes (hospital stay, complications, and vocal cord function assessed via indirect laryngoscopy in case of persistent hoarseness after 3 months of surgery).

### Outcome Measures and Learning Curve Analysis

The primary outcome was the learning curve assessment based on operative time and Cumulative Sum (CUSUM) analysis, which was used to identify the transition points between different learning phases. The cases were divided into phase 1 (initial learning cases) and phase 2 (proficiency cases) based on CUSUM inflection points. Outcomes (incidence of postoperative complications, including transient and permanent RLN injury, seroma, inadvertent parathyroidectomy, rate of conversion to open surgery, and postoperative hospital stay) were compared between the two phases.

### Statistical Analysis

Descriptive statistics was used to summarize the baseline characteristics. Continuous variables (eg, operative time, hospital stay) were compared between phase 1 and phase 2 using *t* test for independent samples. Categorical variables (eg, conversion to open surgery, complications) were analyzed using the chi‐square test or Fisher's exact test, as appropriate. Statistical significance was set at *P* < .05. The learning curve was assessed using CUSUM analysis, and a moving average curve was used to illustrate changes in operative time across consecutive cases.

All statistical analyses were performed using MedCalc software (https://www.medcalc.org/).

## Results

### Patient Characteristics and Outcomes

A total of 103 patients underwent hemithyroidectomy via TOETVA without IONM during the study period; their details are shown in Table 1. The mean age of the patients was 40 ± 8 years, with a slightly higher mean age in phase 2 (41 ± 7 years) compared to phase 1 (38 ± 11 years) (*P* = .045). The male‐to‐female ratio in the entire cohort was 14:89, with no significant difference between the two phases (*P* = .058). The mean nodule size was significantly larger in phase 2 compared to phase 1 (4.5 ± 2.3 cm vs 3.0 ± 1.0 cm; *P* = .003). Tumor localization was predominantly on the right side (79 cases, 76.7%), with no significant difference between the two phases (*P* = .775). Overall, 12 patients had diabetes and 15 had hypertension.

**Table 1:** Patient Characteristics and Comparison of Outcomes of Two Phases

Patient factors	Total cases (n = 103)	Phase 1 (1‐50)	Phase 2 (51‐103)	*P* value
Age (mean ± SD)	40 ± 8	38 ± 11	41 ± 7	.045
Sex (male/female)	14:89	3:47	11:42	.058
Nodule size (mean ± SD, cm)	4.2 ± 1.8	3.0 ± 1	4.5 ± 2.3	.003
Tumor localization (n)				.775
Left	24	9	15	
Right	79	25	54	
Operation type (n)				
Hemithyroidectomy	103	50	53	
Operation time (mean ± SD, min)	144 ± 20	185 ± 24	105.0 ± 12.95	*P* < .001
RLN identification, %	103	50	53	
Conversion to open surgery (n, %)	5	4	1	.325
Outcomes and complications				
Seroma	9	4	5	1.00
Seroma requiring aspiration	3	2	1	
Surgical site infection	2	1	1	
Hoarseness	8	4	4	1.00
Inadvertent parathyroidectomy	1	1	0	
Permanent RLN palsy	0	0	0	
Final histopathology (n)				
Benign	92	48	44	
Malignant	11	02	09	
Postoperative hospital stay (mean ± SD, d)	3 ± 1.8	4 ± 1.5	3 ± 1.0	*P* < .001

Abbreviation: RLN, recurrent laryngeal nerve.

The mean operative time decreased significantly from 185 ± 24 minutes in phase 1 to 105 ± 12.95 minutes in phase 2 (*P* < .001). All cases were successfully completed using the transoral endoscopic approach, with only five patients (4.9%) requiring conversion to open surgery, occurring more frequently in phase 1 (four cases) compared to phase 2 (one case) (*P* = .325). The reason for conversion was hemorrhage obscuring operative view (n = 4) and retrotracheal extension of nodule (n = 1). The incidence of hoarseness and seroma was similar between the two phases (*P* = 1.00). The mean postoperative hospital stay significantly decreased from 4 ± 1.5 days in phase 1 to 3 ± 1.0 days in phase 2 (*P* < .001). Eleven patients were diagnosed with malignancy postoperatively. The majority of patients (n = 6) had a tumor size less than 4 cm and hence are under observation. Four patients underwent open completion thyroidectomy, and one patient underwent trans‐axillary endoscopic completion thyroidectomy.

### Learning Curves for TOETVA

**Figure 1:**
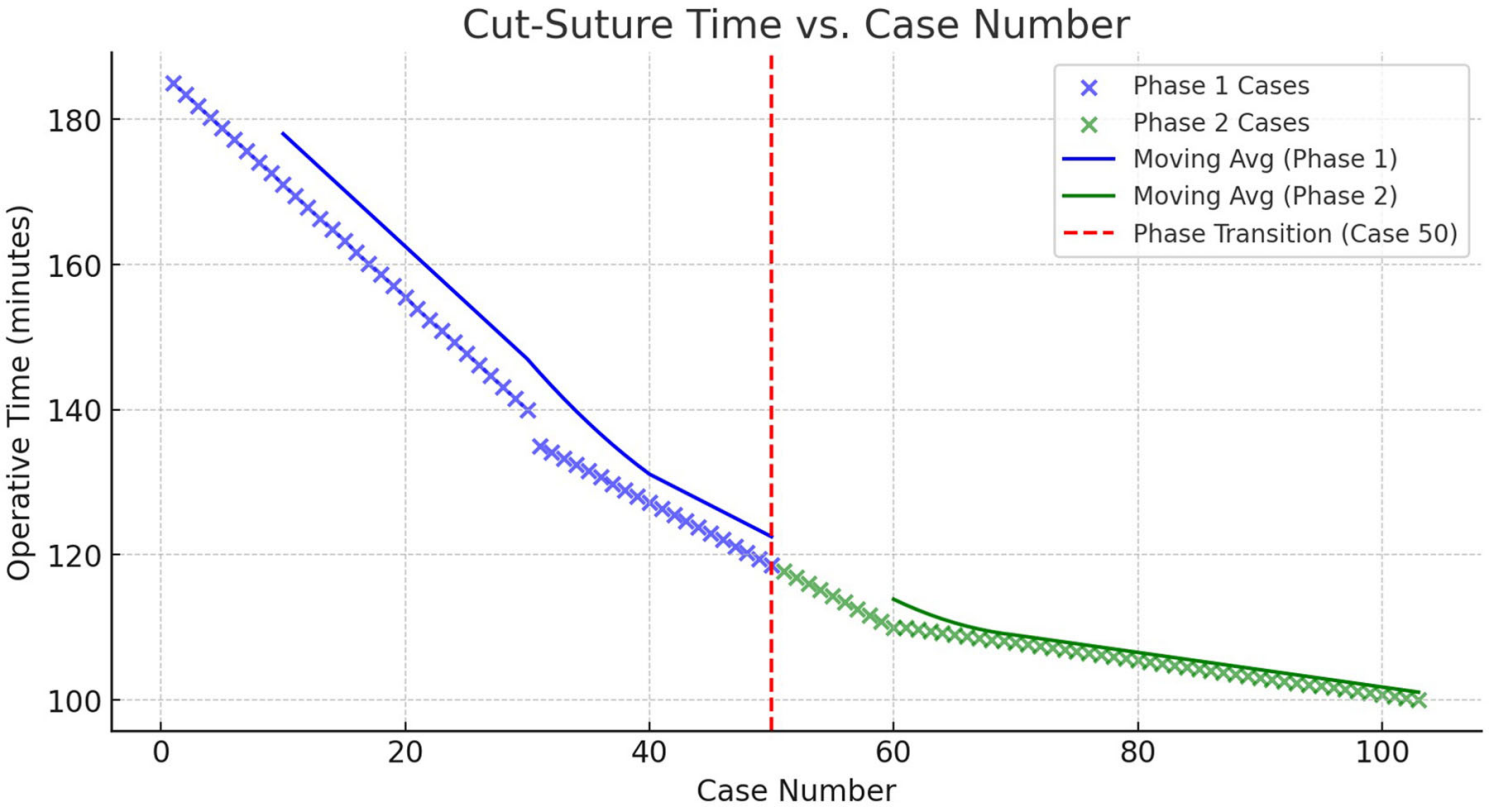
Graph illustrating the operative time versus case number. Phase 1 (blue markers): initial learning phase. Phase 2 (green markers): proficiency phase. Moving average trendlines indicate the rate of improvement.

**Figure 2:**
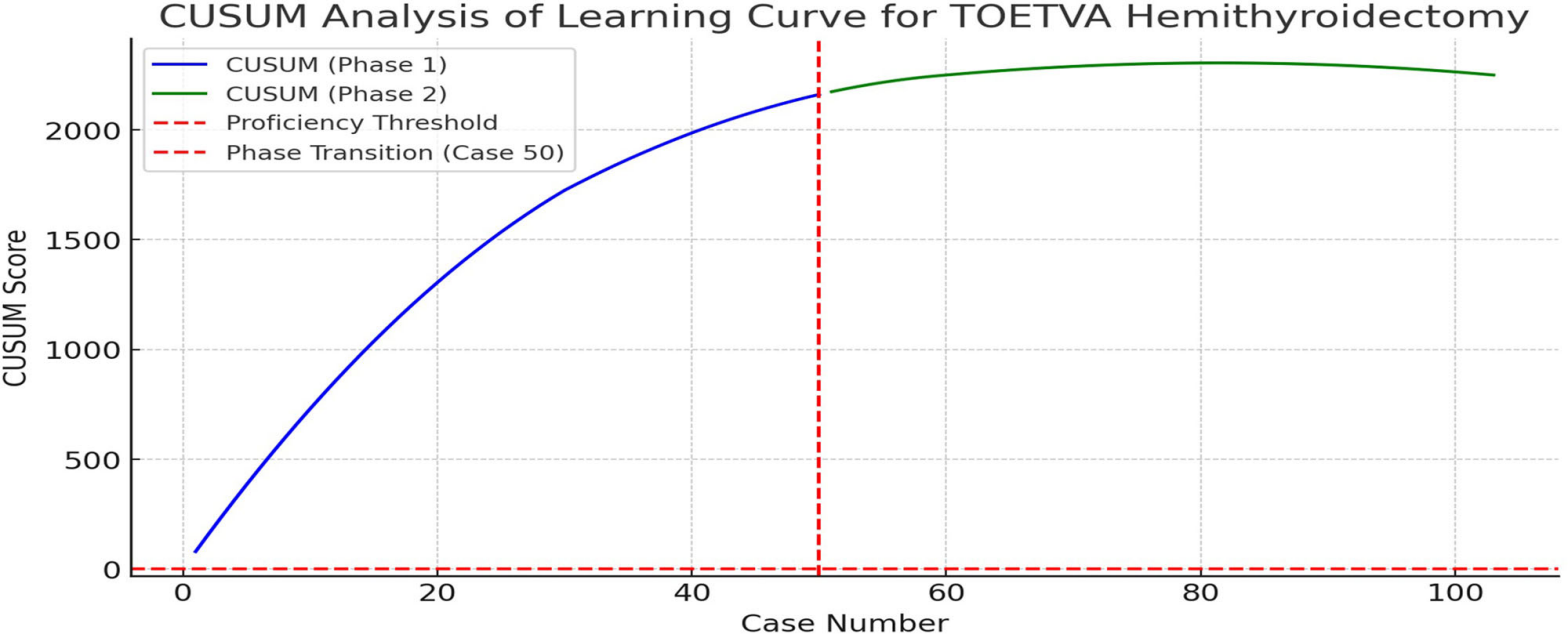
The Cumulative Sum (CUSUM) curve illustrating the learning curve progression: phase 1 (blue line) indicating prolonged operative times, red dashed line the transition point to proficiency.

The learning curve for TOETVA without IONM was assessed using operative time trends and CUSUM analysis, as shown in Figures 1 and 2.

### Operative Time Versus Case Number

Figure 1 illustrates the operative time (cut‐suture time) in relation to the case number, with a distinction between phase 1 (initial learning: cases 1‐50) and phase 2 (proficiency: cases 51‐103). A steep decline in operative time was observed, with cases starting at ~185 minutes and gradually reducing to ~140 minutes by case 50. After case 50, the operative time further decreased and stabilized within 90 to 120 minutes, with a mean of 105 minutes in later cases. The moving average trend lines showed a rapid improvement in phase 1, followed by a plateau in phase 2.

### CUSUM Analysis of Learning Curve

Figure 2 represents CUSUM analysis, which tracks cumulative deviations in operative time from a predefined proficiency threshold (105 minutes). A continuous rise in the CUSUM score was seen in phase 1, reflecting prolonged operative times and high variability.

Transition point at case 50 denotes operative time's stabilization, and the CUSUM curve begins to plateau. In phase 2, the curve flattened and slightly declined, indicating that operative times have reached a stable proficiency level.

## Discussion

This is the first study to evaluate the learning curve for TOETVA without IONM from low‐ and middle‐income countries (LMICs) and shows that TOETVA can be safely performed without IONM with a well‐defined learning curve.

Measuring the surgical learning curve has potential benefits for patient safety and surgical education and can help surgeons to more rapidly achieve/maintain a high expertise level and facilitate the investigators to scientifically report, audit, or research the surgical learning.^17^ We used a CUSUM chart to monitor shifts in the measurable outcome like operative duration as it allows an accurate assessment of the individual's learning curve. Operative time declined steeply from ~185 minutes to ~140 minutes over the first 50 cases, marking the initial learning curve with high variability, before stabilizing at 90 to 120 minutes (mean 105) in later cases, as trend lines indicated rapid improvement in phase 1 followed by a plateau in phase 2, signifying consistency after 50 cases (Figure 2).

Our study's learning curve aligns with global trends,^18^, ^19^, ^20^, ^21^, ^22^, ^23^, ^24^ showing a significant decline in operative time after 50 cases, with proficiency reached thereafter. Uniquely, our cohort had a significantly larger mean nodule size (4.2 ± 1.8 cm) than most prior studies (2‐3 cm) (Table 2), demonstrating that TOETVA can be safely performed for larger nodules. Adhering to the core principles of TOETVA was the key to a safe and successful learning curve.^14^ Adaptation strategies—such as preoperative ultrasound‐guided hydrodissection, a low‐cost indigenous retractor for strap muscles, visual RLN identification, and cost‐effective specimen retrieval (made from hand gloves)—enable this procedure even in resource‐constrained settings.^15^, ^16^ Notably, our proficiency‐phase operative time (105 ± 12.95 minutes) matches studies using IONM, reinforcing that visual RLN identification is reliable in experienced hands.^18^, ^19^, ^23^

**Table 2:** Summary of Studies on Learning Curve

Study	Country	Mean/median nodule size	Mean/median operative time, min	Learning curve (cases to proficiency)	Method used for learning curve analysis	Use of intraoperative neuromonitoring (IONM)	Permanent recurrent laryngeal nerve palsy
Chai et al^18^	South Korea	1.0 ± 0.7 cm	168.0 ± 63.4 (total), 111.0 ± 27.7 (lobectomy)	58	CUSUM	Yes	00
Chai et al^19^	Taiwan	6.06 versus 3.32 cm³ (volume)	144.2 versus 114.2	35	CUSUM	Yes	00
Lira et al^20^	Brazil	Not specified	167‐117	15	Not specified	Not specified	00
Bellini et al^21^	China	﻿1.47‐1.21 cm	147.99 ± 43.67	40‐50	CUSUM	Not specified	00
Qu et al^22^	China	2.4 ± 0.9 cm	﻿113.5 ± 20.1 (learning phase) versus 78.9 ± 16.4 (proficiency phase)	20	Not specified	Not specified	00
Razavi et al^23^	United States	﻿3.3 [0.8‐7.1] cm	191 versus 119	11	Moving average regression	Yes	00
Yu et al^24^	China	﻿6.84 ± 1.84 mm	132.90 versus 123.38	31	CUSUM	Not specified	00
Present study	India	4.5 ± 2.3 versus 3.0 ± 1.0 cm	185 ± 24 versus 105 ± 12.95	50	CUSUM	Not used	00

Abbreviation: CUSUM, Cumulative Sum.

Our study found a low conversion rate to open surgery (4.9%), consistent with previous TOETVA reports.^11^, ^13^, ^25^ The conversion to open surgery occurred predominantly during the initial learning phase (four out of five cases). The major reasons included intraoperative hemorrhage obscuring visualization and retrotracheal nodule extension. These findings underscore the importance of case selection during the early learning curve. As proficiency improved, the rate of conversion significantly declined, indicating that surgeon experience plays a pivotal role in managing intraoperative challenges endoscopically. Hoarseness and seroma rates remained stable across the two learning phases (*P* = 1.00), indicating that the absence of IONM did not increase nerve‐related complications. Notably, postoperative hospital stay decreased from 4 ± 1.5 days in phase 1 to 3 ± 1.0 days in phase 2 (*P* < .001), reflecting improved surgical efficiency and faster recovery with experience. The observed postoperative length of stay was slightly longer than reported in Western series. This may be attributed to multiple factors, including institutional protocols that require patients to stay until public‐funded insurance is approved, as well as socioeconomic factors wherein patients prefer inpatient monitoring before returning home after traveling long distances. Additionally, the initial phase involved a more cautious approach with extended monitoring during the early learning period. A subset of patients (n = 11) diagnosed with thyroid carcinoma postoperatively on final histopathology was safely managed with individualized treatment based on risk stratification and patient preference. Among these, six patients were kept under active surveillance as per current low‐risk thyroid cancer guidelines. Four patients underwent open completion thyroidectomy, whereas one underwent a trans‐axillary endoscopic approach for completion.

Our study has several limitations. First, as a single‐center, single‐surgeon study, its findings may not be generalizable to institutions with different surgical expertise, patient populations, or resource availability. The learning curve and outcomes may vary across centers with diverse training backgrounds and equipment access, and a multicenter study would offer a more comprehensive evaluation of TOETVA without IONM. Second, although our study confirms the safety of TOETVA without IONM, it lacks a direct comparison with IONM‐assisted cases. Additionally, although we highlight TOETVA's feasibility in low‐resource settings, challenges such as instrumentation constraints and procedural modifications may have influenced outcomes and warrant further investigation. We also acknowledge the possibility of early caution during the initial learning phase, which is common in any surgical adoption process. Nonetheless, all patients who met predefined eligibility criteria (benign nodules ≤6 cm, no prior surgery or radiation, etc.) were offered TOETVA regardless of anticipated technical difficulty. These criteria were applied consistently throughout the study. Also, data on patients' body mass index (BMI) were not systematically collected for this retrospective analysis. However, based on clinical observation, the majority of our patients had a relatively lower BMI compared to Western populations, which may have facilitated better exposure and dissection during TOETVA. BMI is an important factor in transoral surgery, and future prospective studies should explore its role in predicting outcomes and optimizing patient selection. Lastly, in our setting, due to resource constraints and the high‐volume nature of our public hospital, laryngoscopy to assess RLN was performed only in patients with persistent hoarseness beyond the immediate postoperative period.

Despite its limitations, our study is the first large‐cohort analysis from an LMIC to evaluate TOETVA without IONM, which reflects the real‐world scenario in most resource‐limited regions. IONM, although useful, is not universally accessible or affordable, and our findings validate the feasibility and safety of TOETVA when performed without it. Additionally, our cohort had significantly larger nodules (mean size 4.5 ± 2.3 cm in phase 2) compared to most previous studies, and we introduced several low‐cost technique adaptations (eg, indigenous retractors, glove‐based specimen retrieval), reinforcing the value of our work in LMICs. Lastly, the CUSUM‐based learning curve analysis offers an objective, detailed assessment of surgical proficiency milestones, providing a valuable reference for training programs in emerging TOETVA centers. This has important implications for resource‐limited settings where the availability of IONM remains a significant barrier to adopting this technique. The ability to achieve comparable safety and efficiency outcomes without IONM broadens the feasibility of this approach in LMICs.

## Conclusion

Our study demonstrates that TOETVA can be safely performed without IONM, with a well‐defined learning curve. Operative time and hospital stay significantly decreased after 50 cases, marking it the proficiency threshold.

## Author Contributions

**Sanjay Kumar Yadav**, Conceptualization; methodology; data collection; statistical analysis; manuscript drafting; data validation; manuscript review; critical revisions. **Goonj Johri**, Data validation; manuscript review; critical revisions. **Saket Shekhar**, Statistical analysis. **Pawan Agarwal**, Data validation; manuscript review; critical revisions. **Dhananjaya Sharma,** Supervision; final manuscript approval.

## Disclosures

### Competing interests

Sanjay Kumar Yadav, Goonj Johri, Saket Shekhar, Pawan Agarwal, and Dhananjaya Sharma have no conflicts of interest to disclose.

### Funding source

Sanjay Kumar Yadav, Goonj Johri, Saket Shekhar, Pawan Agarwal, and Dhananjaya Sharma have no financial ties to disclose.

## Data Availability

The data sets generated and analyzed during the study are available from the corresponding author upon reasonable request.
